# RGS5 maintaining vascular homeostasis is altered by the tumor microenvironment

**DOI:** 10.1186/s13062-023-00437-y

**Published:** 2023-11-20

**Authors:** Peng Kong, Xu Wang, Ya-Kun Gao, Dan-Dan Zhang, Xiao-Fu Huang, Yu Song, Wen-Di Zhang, Rui-Juan Guo, Han Li, Mei Han

**Affiliations:** 1 Department of Biochemistry and Molecular Biology, College of Basic Medicine, Key Laboratory of Neural and Vascular Biology of Ministry of Education, Key Laboratory of Medical Biotechnology of Hebei Province, Hebei Medical University, Shijiazhuang, China; 2 Department of Pathology, The Fourth Hospital of Hebei Medical University, Hebei Medical University, Shijiazhuang, China; 3 Department of Orthopaedic Surgery, Institute of Biomechanical Science and Biomechanical Key Laboratory of Hebei Province, Third Hospital of Hebei Medical University, Shijiazhuang, China

**Keywords:** Vascular smooth muscle cells, Regulator of G protein signaling 5, Vascular remodeling, Breast cancer

## Abstract

**Background:**

Regulator of G protein signaling 5 (RGS5), as a negative regulator of G protein-coupled receptor (GPCR) signaling, is highly expressed in arterial VSMCs and pericytes, which is involved in VSMC phenotypic heterogeneity and vascular remodeling in tumors. However, its role in normal and tumor vascular remodeling is controversial.

**Methods:**

RGS5 knockout (*Rgs5*-KO) mice and RGS5 overexpression or knockdown in VSMCs in vivo by adeno-associated virus type 9 (AAV) carrying RGS5 cDNA or small hairpin RNA (shRNA) targeting RGS5 were used to determine the functional significance of RGS5 in vascular inflammation. RGS5 expression in the triple-negative (TNBCs) and non-triple-negative breast cancers (Non-TNBCs) was determined by immunofluorescent and immunohistochemical staining. The effect of breast cancer cell-conditioned media (BC-CM) on the pro-inflammatory phenotype of VSMCs was measured by phagocytic activity assays, adhesion assay and Western blot.

**Results:**

We identified that knockout and VSMC-specific knockdown of RGS5 exacerbated accumulation and pyroptosis of pro-inflammatory VSMCs, resulting in vascular remodeling, which was negated by VSMC-specific RGS5 overexpression. In contrast, in the context of breast cancer tissues, the role of RGS5 was completely disrupted. RGS5 expression was increased in the triple-negative breast cancer (TNBC) tissues and in the tumor blood vessels, accompanied with an extensive vascular network. VSMCs treated with BC-CM displayed enhanced pro-inflammatory phenotype and higher adherent with macrophages. Furthermore, tumor-derived RGS5 could be transferred into VSMCs.

**Conclusions:**

These findings suggest that tumor microenvironment shifts the function of RGS5 from anti-inflammation to pro-inflammation and induces the pro-inflammatory phenotype of VSMCs that is favorable for tumor metastasis.

**Supplementary Information:**

The online version contains supplementary material available at 10.1186/s13062-023-00437-y.

## Introduction

Vascular smooth muscle cells (VSMCs) play a critical role in controlling vascular homeostasis, as well as maintaining vascular structural integrity and vascular remodeling. As a necessary corollary of role in tissue homeostasis and repair, VSMCs have considerable phenotypic plasticity and can dedifferentiate from a contractile to a synthetic state or transdifferentiate into macrophage-like cells, contributing to the protective or deleterious effects in response to vascular injury or disease [1]. Regulator of G protein signaling 5 (RGS5), as a negative regulator of G protein-coupled receptor (GPCR) signaling, is highly expressed in arterial VSMCs and pericytes [2, 3], which regulates arterial tone and blood pressure [4, 5]. VSMC phenotypic heterogeneity is also regulated by the epigenetic programs that control RGS5 expression [6]. RGS5 has been shown to regulate inflammation-related signaling pathways that shape tissue damage and fibrosis, which is involved in vascular remodeling in tumors [7], hypertension [8, 9] and atherosclerosis [10–12]. However, the role of RGS5 in regulation of VSMC phenotypic switching is controversial.

The previous studies have demonstrated that overexpressed RGS5 attenuates the proliferation and migration of VSMCs to prevent vascular remodeling and neointima formation [13, 14]. We recently identify that RGS5 inhibits Sox10 phosphorylation and activation, resulting in blockade of VSMC transdifferentation and reduction vascular inflammation [15]. Conversely, elevated levels of RGS5 expression are demonstrated to be strongly correlated with active tumor vessel remodeling during carcinogenesis. Remarkably, tumors arising in a RGS5-deficient background display vessels with normalized morphology and an overall improved blood flow [16], indicating that RGS5 contributes to tumor vascular remodeling. As can be deduced from these divergent results, the functional role of RGS5 in the regulation of vascular remodeling may be affected by different microenvironmental factors.

In this study, we demonstrated that RGS5 ablation promoted the pro-inflammatory phenotypic switching of VSMCs and vascular hyperplasia via induction of pyroptosis and local immune dysregulation. Overexpression of RGS5 inhibited vascular inflammation and vascular remodeling. However, in the context of breast cancer tissues, the role of RGS5 was completely disrupted, which mediated VSMC inflammation and vascular remodeling in the tumor tissues. These findings identify a novel mechanism of vascular remodeling to support tumor growth and survival, shifting from an antagonistic to pro-inflammatory RGS5.

## Materials and methods

### Animals

All animal experiments were approved by the Institutional Animal Care and Use Committee of Hebei Medical University. C57BL/6 J wild-type (WT) mice were purchased from Charles River. *RGS5* knockout (*RGS5*-KO) mice were kindly gifted by Institute of Model Animal of Wuhan University, China. *SM22α*-KO mice were purchased from the Jackson Laboratory. All animals were housed in a pathogen-free environment with the ambient temperature maintained at 21℃ to 23℃ and relative humidity at 50% to 60%, with a 12 h: 12 h light: dark cycle. Animals were allowed ad libitum access to water and food unless otherwise indicated.

### Human breast cancer tissues

Human breast cancer samples were obtained from 50 patients undergoing surgery at the Fourth Hospital of Hebei Medical University (Shijiazhuang, China). The Ethical Committee of Hebei Medical University approved all protocols using human samples. All patients or their relatives provided written informed consent prior to their participation in the study (2022KS023).

### Carotid artery ligation model and oligonucleotide treatment of mice

Ten- to twelve- week-old male mice were anaesthetized using 2.5–3% isoflurane by inhalation. The left common carotid artery was tied firmly with one knot using 6–0 silk suture just below the bifurcation point. In sham (unligated) animals, the suture was passed under the exposed left carotid artery but not tightened. For adeno-associated virus (AAV) treatment, 5 × 10^10^ pfu/ml adeno-associated virus (AAV-CON, AAV-shRGS5 or AAV-RGS5) was suspended in 100 μL F-127 pluronic gel (Sigma-Aldrich; 25% wt/vol) and applied around the carotid artery, respectively. Carotid arteries were harvested at indicated time points after ligation. All procedures were performed by a single operator to attain a constant degree of vessel wall injury for each of the animals. This study was reviewed and approved by the Institutional Animal Care and Use Committee at Hebei Medical University.

### Hematoxylin and eosin (H&E) staining

The common carotid arteries of mice and human breast tissues were fixed with 4% paraformaldehyde, then embedded in paraffin. Serial Sects. (8 μm thick) were obtained at 500 μm proximal to the ligation site. These sections were stained with hematoxylin and eosin (Leica Biosystems). Image ProPlus 6.0 software (Media Cybernetics) was performed to measure the cross-sectional areas of the neointima, the media, artery lumen diameter, artery lumen area and external elastic lamina (EEL) circumference by a single-name observer in a blinded manner. A mean value was determined for each animal from at least three sections.

### Histological analyses

For immunohistochemical staining, frozen sections were incubated with 3% hydrogen peroxide, followed by blocking with 3% normal blocking serum. The sections were incubated with primary antibodies against RGS5 (1:100, Proteintech), SM α-actin (1:50, Proteintech) or CD34 (1:50, GeneTex) at 4℃ overnight, followed by a secondary antibody before staining with the DAB Kit (ZSGB-BIO, Beijing, China). Nuclei were counter-stained with hematoxylin. Sections incubated with species-matched IgG alone were used as negative controls.

For Immunofluorescent staining, cells were fixed in 4% paraformaldehyde and permeabilized with 0.1%Triton X-100 at room temperature for 20 min. Thereafter, cells were blocked with 5% goat serum albumin in PBS for 1 h and incubated with primary antibodies at 4℃ overnight. For the tissue samples, the vessels were collected and washed with PBS to remove excessive blood. After being fixed in 4% paraformaldehyde for 20 min, vessels were embedded in OCT (Sakura) and serial Sects. (5 μm thick) were obtained at 500 μm proximal to the ligation site. Cryosections were air-dried for about 30 min at room temperature, following by washing in PBS for three times and incubation with blocking buffer (5% Goat serum) for 30 min at room temperature and then incubated with primary antibody overnight at 4℃. Signals were detected by using Alexa fluorescence-conjugated secondary antibodies (Invitrogen) for 1 h at room temperature. Images were acquired using a laser scanning confocal microscope (Leica SP5, Switzerland). Image acquisition settings were adjusted with unstained samples, and isotype controls were kept throughout the experiment. Quantitative analysis was performed on more than three slices using Image J software.

### Cells culture and treatment

VSMCs isolated from the aorta of the mice were cultured in low glucose Dulbecco’s-modified Eagle’s medium (DMEM) (Invitrogen) supplemented with 20% fetal bovine serum (FBS, Gibco), 100 U/mL penicillin, and 100 μg/mL streptomycin. VSMCs were maintained at 37℃ in a humidified atmosphere containing 5% CO_2_, and only 4–15 passages of VSMCs were used in the experiments. Serum starvation was done by withdrawing serum and incubating the cells in 0.5% FBS for 24 h before stimulating with TNF-α (20 ng/mL), and infection with Lentivirus. Human breast cancer cells MDA-MB-231 and MCF-7 were purchased from ATCC and maintained in low glucose DMEM supplemented with 10% FBS, 100 U/mL penicillin, and 100 μg/mL streptomycin.

For the preparation of BC-CM, briefly, MDA-MB-231 or MCF-7 tumor cells were plated and cultured in 10-cm culture dishes. After 12 h, the medium was replaced with 10 mL serum-free medium. For 48 h, the medium was then collected, filtered and centrifuged.

### Western blot analysis

Lysates from cells or tissue samples were prepared with lysis buffer (Beyotime) and the protein concentrations were determined using the Bradford method. Equal amounts of protein (30–100 μg) were separated by 8% or 10% or 12% SDS-PAGE, and electro-transferred to a PVDF membrane (Merck). After blocking with 5% milk in TBST, the membranes were incubated with primary antibodies against VCAM-1 (1:1000, Epitomics), RGS5 (1:500, Proteintech), MCP-1(1:200, Santa Cruz), C3 (1:250, Abcam), CD74 (1:500, Abcam), Lyz2 (1:200, Proteintech) α-SMA(1:1000, Proteintech), Caspase-1 (1:200, Proteintech), GSDMD (1:200, Abcam) or β-actin (1:1000, Proteintech) at 4℃ overnight, and then with the HRP-conjugated secondary antibody for 1 h. The blots were visualized using Image Quant LAS 4000 detection system (GE Healthcare). Band intensities were quantified with Image Pro Plus 6.0 software.

### RNA isolation and quantitative reverse transcription-PCR (qRT-PCR)

Total RNAs of cell or tissue samples were extracted using TRIzol reagent (Invitrogen) and treated with DNaseI to remove genomic DNA. cDNAs were synthesized using the M-MLV First Strand Kit (Life Technologies), and quantitative PCRs were performed using SYBR Green qPCR SuperMix-UDG (Life Technologies). Relative mRNA expression was normalized to β-actin levels, using the 2^−△△Ct^ method. The average threshold cycle for each gene was determined from at least three independent experiments. The specific primer sequences used for qRT-PCR were listed in Additional file 1: Table S1.

### Phagocytic activity assays

Phagocytic activity assays were performed as described [17]. In brief, cells were seeded in 96-well plates. After conditional treatment, cells were washed with PBS, added 100 mL of 0.1% neutral red solution (Sigma) and incubated for 30 min, washed cells again and added 100 mL of cell-lysis solution (1 M glacial acetic acid/ethanol = 1:1). The plates were incubated at 37℃ for 4 h in a 5% CO_2_ atmosphere. The resulting absorbance was measured at 540 nm.

For fluorescent microspheres assay, cells were seeded on coverslips in 12-well plates. After indicated treatment, the cells were washed with PBS, added 600 mL of 0.25% fluorescent microsphere solution (Sigma) and incubated at 37℃ for 2 h. Then, cells were washed three times with PBS to remove the free microspheres, and fixed with 4% paraformaldehyde. The nuclei were stained with DAPI (Invitrogen) for 15 min. Images were acquired using a Leica microscope (Leica SP5, Switzerland) and digitized with a software program LAS AF Lite.

### Adhesion assays

VSMCs plated in 96-well culture plates were treated with BC-CM or not, and then, RAW264.7 cells labeled with calcein-AM (Life Technologies) were added to each well. After 30 min incubation, non-adherent cells were removed carefully by washing with cold phosphate-buffered saline (PBS). The fluorescent intensities were determined by excitation and emission at 490 and 535 nm, respectively.

### siRNA transfection

The siRNA duplexes targeting RGS5 (si-RGS5), 5’-GGGUUGCCUGUGAGAAUUATT-3’ and 5’-UAAUUCUCACAGGCAACCCTT-3’ were obtained from GenePharma. Scrambled siRNA (si-Con) 5’-UUCUCCGAACGUGUCACGUTT-3’ and 5’-ACGUGACACGUUCGGAGAATT-3’ served as a negative control. The cultured VSMCs were grown to 50–60% confluence and then transfected with siRNA using HighGene transfection reagent (ABclonal) according to the manufacturer’s instructions.

### Viral gene delivery

For VSMC-specific *Rgs5* deletion and overexpression, the Adeno-associated virus AAV-shRGS5 or AAV-RGS5 was constructed and packaged by Hanbio Biotechnology (Shanghai, China). Specific shRNA against RGS5 with a SM22α promoter and non-targeting control shRNA were separately constructed in the same vector, and AAV serotype 9 viral particles harboring these sequences were generated. The targeted sequences for AAV are 5’-GGGUUGCCUGUGAGAAUUATT-3’ and 5’-UAAUUCUCACAGGCAACCCTT-3’, respectively. For VSMC-specific RGS5 overexpression, the same serotype AAV containing a cDNA sequence specific for murine RGS5 or negative control was constructed and packaged by Hanbio Biotechnology (Shanghai, China). The AAV was injected via the tail vein with a standard dose 5 × 10^9^ pfu/kg. The tail was cleansed with 70% ethanol and the injection was made in the lateral vein, using 30- gauge needles. After 21 days, the mice were randomly grouped for subsequent experiments.

### Statistical analysis

Statistical analysis was performed using GraphPad Prism 8.0 software (GraphPad Software, San Diego, CA, USA). Data are presented as the means ± SD from at least three independent experiments (n ≥ 3), and each independent experiment was repeated three times to obtain the mean. For data, Shapiro–Wilk was used to test normality. Differences between two groups were compared by Student’s t tests. In all cases, statistical significance was concluded where the two-tailed probability was less than 0.05.

## Results

### RGS5 ablation promotes VSMC inflammatory response and vascular remodeling

**Fig. 1:**
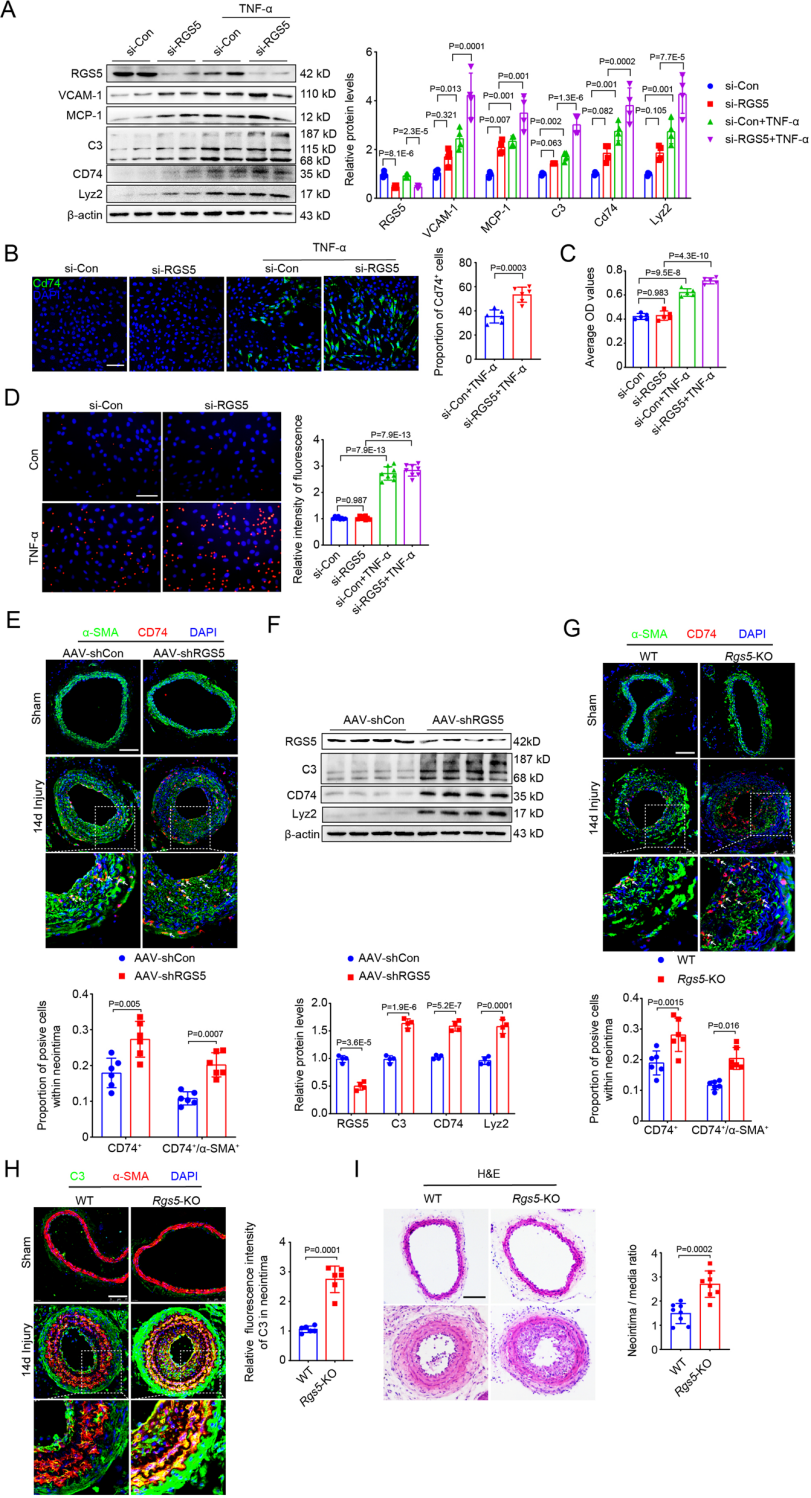
RGS5 ablation promotes VSMC inflammatory response and vascular remodeling. (**A**) Western blot analysis for RGS5, inflammatory markers (MCP-1 and VCAM-1) and macrophage-like markers (C3, CD74 and Lyz2) in mouse VSMCs transfected with si-Con (50 nM) or si-RGS5 (50 nM) followed TNF-α treatment for 48 h. (**B**) Representative immunofluorescence images of CD74 (green) stained in mouse VSMCs transfected with siRNA (si-Con) (50 nM) or siRNA-RGS5 (si-RGS5) (50 nM) followed TNF-α treatment for 72 h. Nucleuses stained by DAPI were indicated in blue. Scale bar = 75 μm. (**C**) Phagocytosis of neutral red by VSMCs transfected with si-Con (50 nM) or si-RGS5 (50 nM) followed TNF-α treatment for 7 days. (**D**) Phagocytosis of fluorescent microsphere by VSMCs transfected with si-Con (50 nM) or si-RGS5 (50 nM) followed TNF-α treatment for 7 days. (**E, G**) Representative immunofluorescence images of CD74 and α-SMA in sham-operated and ligated carotid arteries at day 14 post surgery of 10-week-old male WT mice intravenous infected with AAV-shRGS5 or 10-week-old male *Rgs5*-KO mice and WT littermates. Scale bar = 75 µm. (**F**) Western blot analysis and quantification of protein expression of RGS5 and macrophage-like markers (C3, CD74 and Lyz2) in lysates of carotid arteries harvested from 10-week-old male WT mice intravenous infected with AAV-shRGS5 following by carotid artery ligation for 14 days. (**H, I**) Representative cross sections of immunofluorescence or hematoxylin & eosin-staining (H&E) images of C3 and α-SMA in sham-operated and ligated carotid arteries at day 14 post surgery of 10-week-old male *Rgs5*-KO mice and WT littermates. Scale bar = 75 µm. The data were presented as the mean ± SD

To determine that RGS5 is causally involved in VSMC inflammatory response, we knocked down the expression of RGS5 in VSMCs in vitro by transfecting siRNA targeting mouse RGS5 (si-RGS5). RGS5 silencing increased the expression of pro-inflammatory markers VCAM-1 and MCP-1 and enhanced TNF-α-induced upregulation of VCAM-1, MCP-1, C3, CD74 and Lyz2 (Fig. 1A). The number of CD74^+^ macrophage-like VSMCs dramatically increased with long-term TNF-α treatment (72 h) compared to the negative control (si-Con) (Fig. 1B). Furthermore, the phagocytic activity was remarkably increased in RGS5 knockdown VSMCs (Fig. 1C, D), further suggesting that downregulation of RGS5 promotes VSMC pro-inflammatory phenotypic switching.

We next validated the positive effect of RGS5 ablation on VSMC pro-inflammatory phenotypic switching in vivo by VSMC-specific knockdown of RGS5 expression using an adeno-associated virus type 9 (AAV) carrying small hairpin RNA (shRNA) targeting RGS5 (AAV-shRGS5) (Additional file 2: Fig. S1A, B). Immunofluorescence staining revealed prominent accumulation of CD74^+^/α-SMA^+^ and CD74^+^/CD68^+^ cells in the neointima of SMC-specific RGS5 knockdown mice after ligation for 14 days, compared to AAV-shCon group (Fig. 1E, Additional file 2: Fig. S1C). Furthermore, RGS5 silencing further elevated the expression of C3, CD74 and Lyz2 proteins in AAV-shRGS5 arteries compared to AAV-shCon neointima (Fig. 1F). The aggravated vascular inflammation was further confirmed in the neointima of global *RGS5* knockout (*Rgs5*-KO) mice, which exhibited increased accumulation of CD74^+^/α-SMA^+^, CD74^+^/CD68^+^ cells and elevated C3 expression, compared with their wild-type (WT) littermates (Fig. 1G, H, Additional file 2: Fig. S1D), accompanied by thicker neointima in *Rgs5*-KO mice (Fig. 1I). Thus, a loss-of-function mutation in RGS5 drives VSMC inflammation and sustained vascular remodeling.

### RGS5 ablation contributes to pyroptosis and vascular inflammation

**Fig. 2:**
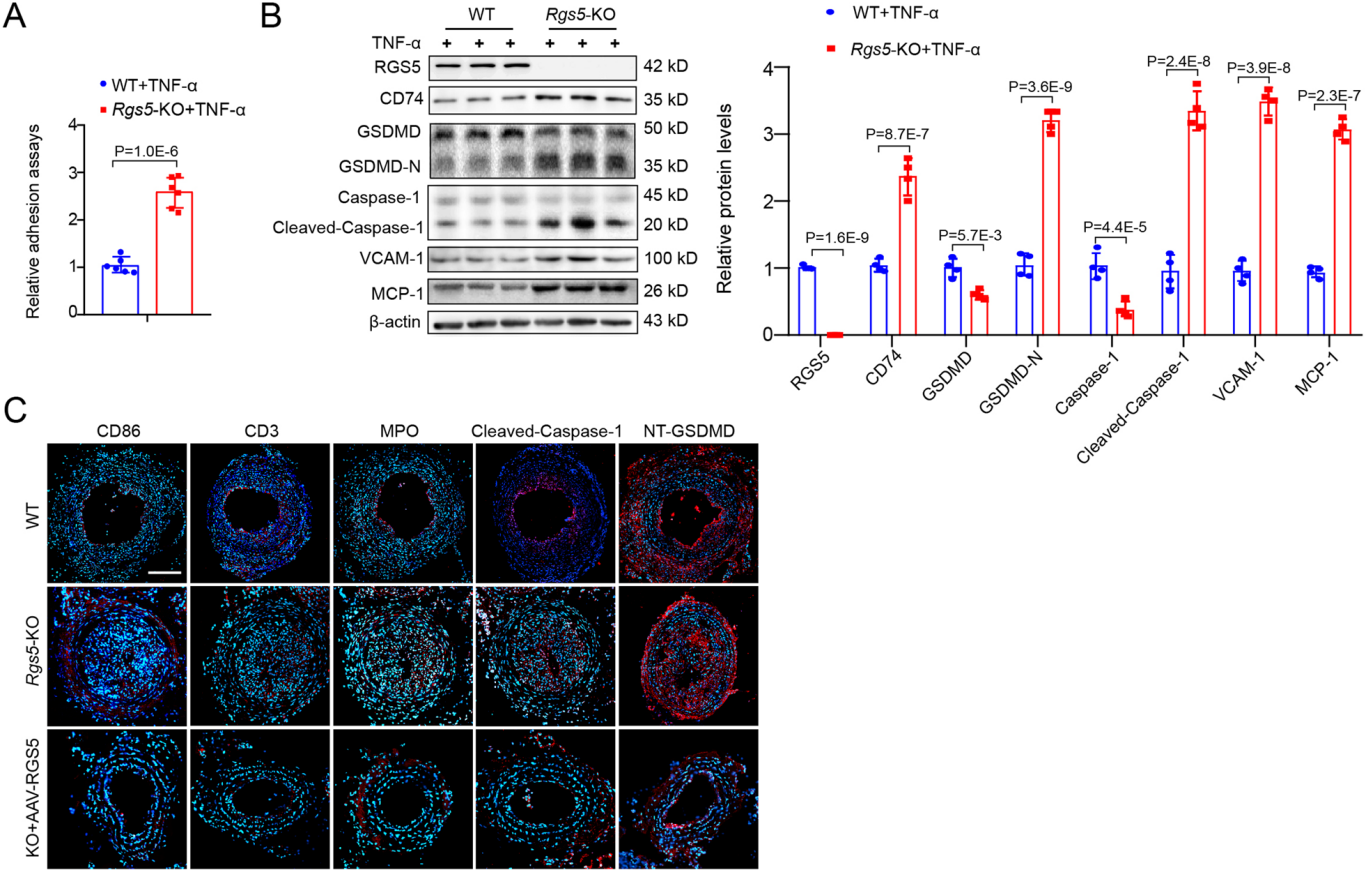
RGS5 ablation contributes to pyroptosis and vascular inflammation. (**A**) The fluorescent intensity quantification of calcein-AM-labeled RAW264.7 cell adhesion to WT and *Rgs5*-KO VSMCs treated with TNF-α. (**B**) Western blot analysis and quantification of RGS5, CD74, GSDMD, GSDMD-N, Caspase-1 and cleaved Caspase-1 in WT and *Rgs5*-KO VSMCs stimulated with TNF-α for 48 h, respectively. (**C**) Representative immunofluorescence images of CD86, CD3, MPO, Cleaved-Caspase-1 and N-terminal of GSDMD (NT-GSDMD) stained in ligated carotid arteries for 14 days of WT or *Rgs5*-KO mice, or *Rgs5*-KO mice with intravenous injection of AAV-Flag-RGS5 (KO + AAV-RGS5) for 4 weeks. Scale bar = 75 μm. The data were presented as the mean ± SD

We next examined the effect of RGS5 ablation-induced inflammation of VSMCs on local immune microenvironment in vascular remodeling. We showed that *Rgs5*-KO VSMCs exhibited enhanced activity of recruitment of macrophages and pyroptosis compared to WT VSMCs in the induction of transdiferentiation context in vitro, as shown by increased adhesion to RAW264.7 cells and elevated expression level of pyroptosis-related genes cleaved-Caspase-1 and GSDMD N-terminal (GSDMD-NT) (Fig. 2A, B). Similarly, accumulation of macrophage-like VSMCs was accompanied by increased infiltration of inflammatory immune cells including T cells (CD3^+^), neutrophils (MPO^+^) and M1 macrophages (CD86^+^) and pyroptosis in neointimal hyperplasia of *Rgs5*-KO mice compared with WT mice in vivo*,* as indicated by increased the GSDMD-NT and cleaved-Caspase-1 staining in the neointima cells (Fig. 2C). Collectively, RGS5 ablation induced VSMC pro-inflammatory phenotype contributes to cell pyroptosis and local immune dysregulation in vascular remodeling.

### RGS5 protects against inflammatory response of VSMCs and ameliorates vascular remodeling

**Fig. 3:**
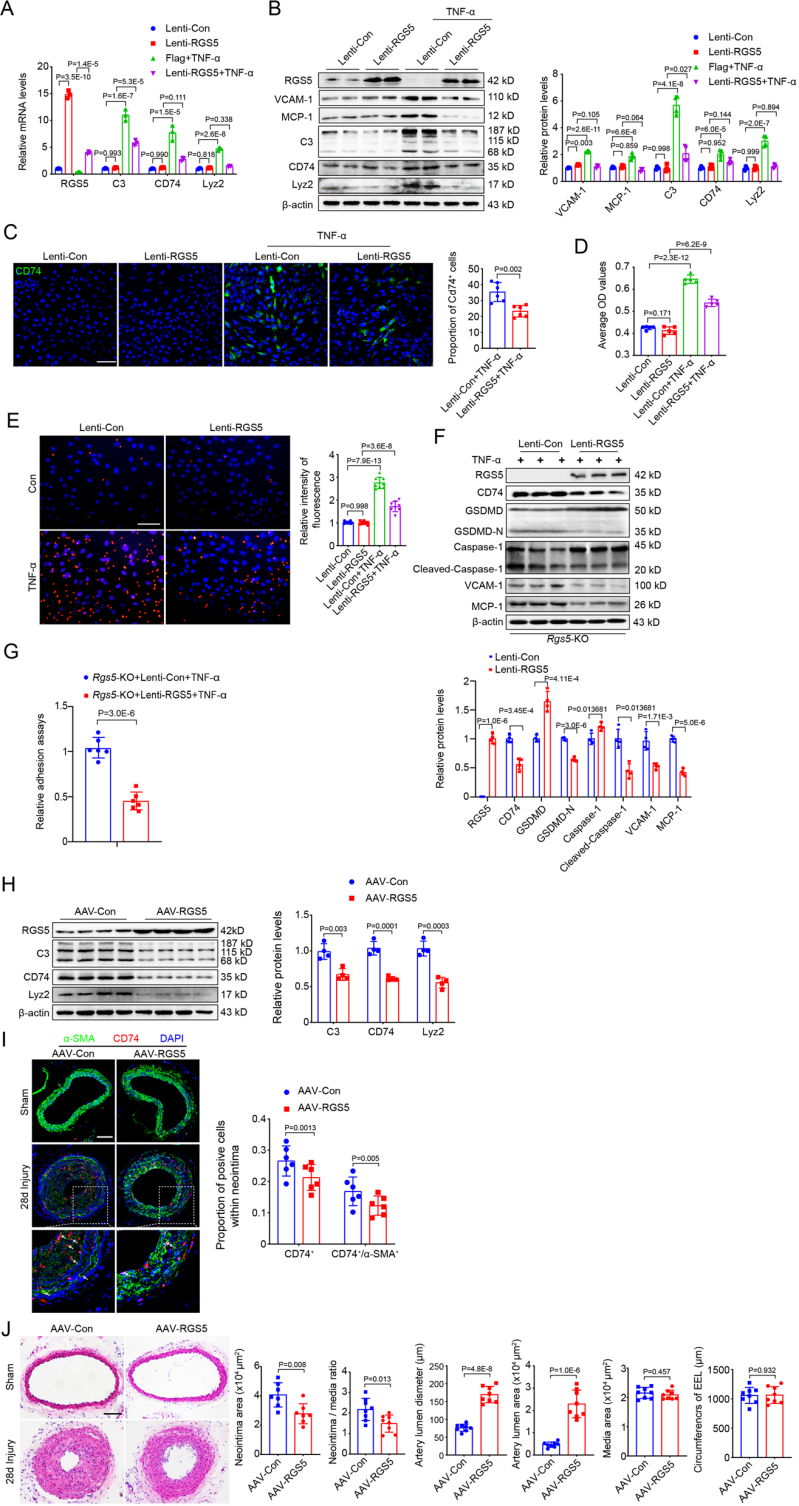
RGS5 protects against inflammatory response of VSMCs and ameliorates vascular remodeling. (**A**) qRT-PCR analysis of RGS5, inflammatory markers (MCP-1 and VCAM-1) and macrophage-like markers (C3, CD74 and Lyz2) mRNA levels in mouse VSMCs with RGS5 overexpression followed TNF-α treatment for 24 h. (**B**) Western blot analysis and quantification of protein expression of RGS5, inflammatory markers (MCP-1 and VCAM-1) and macrophage-like markers (C3, CD74 and Lyz2) in mouse VSMCs infected with lenti-RGS5 followed TNF-α treatment for 48 h. (**C**) Left, Representative immunofluorescence images of CD74 (green) stained in mouse VSMCs infected with lenti-RGS5 followed by TNF-α treatment for 72 h. Nucleuses stained by DAPI were indicated in blue. Scale bar = 75 μm. (**D**) Phagocytosis of neutral red of VSMCs with RGS5 overexpression followed TNF-α treatment for 7 days. (**E**) Phagocytosis of fluorescent microsphere of VSMCs with RGS5 overexpression followed TNF-α treatment for 7 days. (**F**) Western blot analysis and quantification of RGS5, CD74, GSDMD, GSDMD-N, Caspase-1 and cleaved Caspase-1 in *Rgs5*-KO VSMCs infected with lenti-RGS5 followed TNF-α treatment for 48 h. (**G**) The fluorescent intensity quantification of calcein-AM-labeled RAW264.7 cell adhesion to *Rgs5*-KO VSMCs infected with lenti-RGS5 followed TNF-α treatment. (**H**) Western blot analysis RGS5 and macrophage-like markers (C3, CD74 and Lyz2) in lysates of carotid arteries harvested from 10-week-old male WT mice intravenous infected with AAV-Con and AAV-Flag-RGS5 following by carotid artery ligation for 28 days. (**I**) Representative cross sections of immunofluorescence images of CD74 and α-SMA in sham-operated and ligated carotid arteries at day 28 post surgery of 10-week-old male WT mice intravenous infected with AAV-Con and AAV-RGS5. Scale bar = 75 µm. (**J**) Left: Representative cross sections of hematoxylin & eosin-stained (H&E) sham-operated and ligated carotid arteries at day 28 post surgery of 10-week-old male WT mice intravenous infected with AAV-Con and AAV-RGS5. Scale bar = 100 µm. Right: Quantitative analysis of the intima area, intima to media ratio, artery lumen diameter, artery lumen area, external elastic lamina (EEL) circumference and media area in histological sections from ligated carotid arteries of 10-week-old male WT mice at day 28 post surgery of 10-week-old male WT mice intravenous infected with AAV-Con and AAV-RGS5. The data were presented as the mean ± SD

We next investigated whether RGS5 could inhibit the pro-inflammatory phenotypic switching of VSMCs by transfecting lentiviruses encoding the full-length RGS5 transcript (Lenti-RGS5). The gain-of-function experiments revealed that overexpression of RGS5 dampened TNF-α-induced expression of the adhesion molecules and pro-inflammatory markers (Fig. 3A, B). Immunofluorescence staining showed the reduced number of CD74^+^ cells (Fig. 3C) and attenuated phagocytic activity in RGS5-overexpressed VSMCs compared to the vehicle-treated cells (Fig. 3D, E), representing a reduced pro-inflammatory phenotype of VSMCs. Importantly, rescue of RGS5 expression reversed increased pyroptosis and macrophage recruitment in *Rgs5*-KO VSMCs (Fig. 3F, G), which may be associated with reduced expression of VCAM-1 and MCP-1 that play a major role in the cascade of immune cell transmigration into tissues [18].

To determine RGS5 inhibiting VSMC inflammation in vivo, we constructed mice with VSMC-specific RGS5 overexpression mediated by AAV carrying RGS5 cDNA driven by the *Sm22α* promoter (Additional file 2: Fig. S2A). AAV-mediated RGS5 transgenic delivery dramatically increased RGS5 expression and abolished up-regulation of macrophage-like marker expression (Fig. 3H), which decreased the number of CD74^+^/α-SMA^+^ and CD74^+^/CD68^+^ cells in the neointimal hyperplasia of mice (Fig. 3I, Additional file 2: Fig. S2B). In agreement, administration of AAV-RGS5 remarkably attenuated the development of vascular remodeling, as indicated by reduced neointima thickness (Fig. 3J). Together, these results suggest that RGS5 offers a protective effect on vascular homeostasis via blocking VSMC inflammation and pyroptosis.

### SM22α acts additively and synergistically with RGS5 to suppress VSMC inflammation

**Fig. 4:**
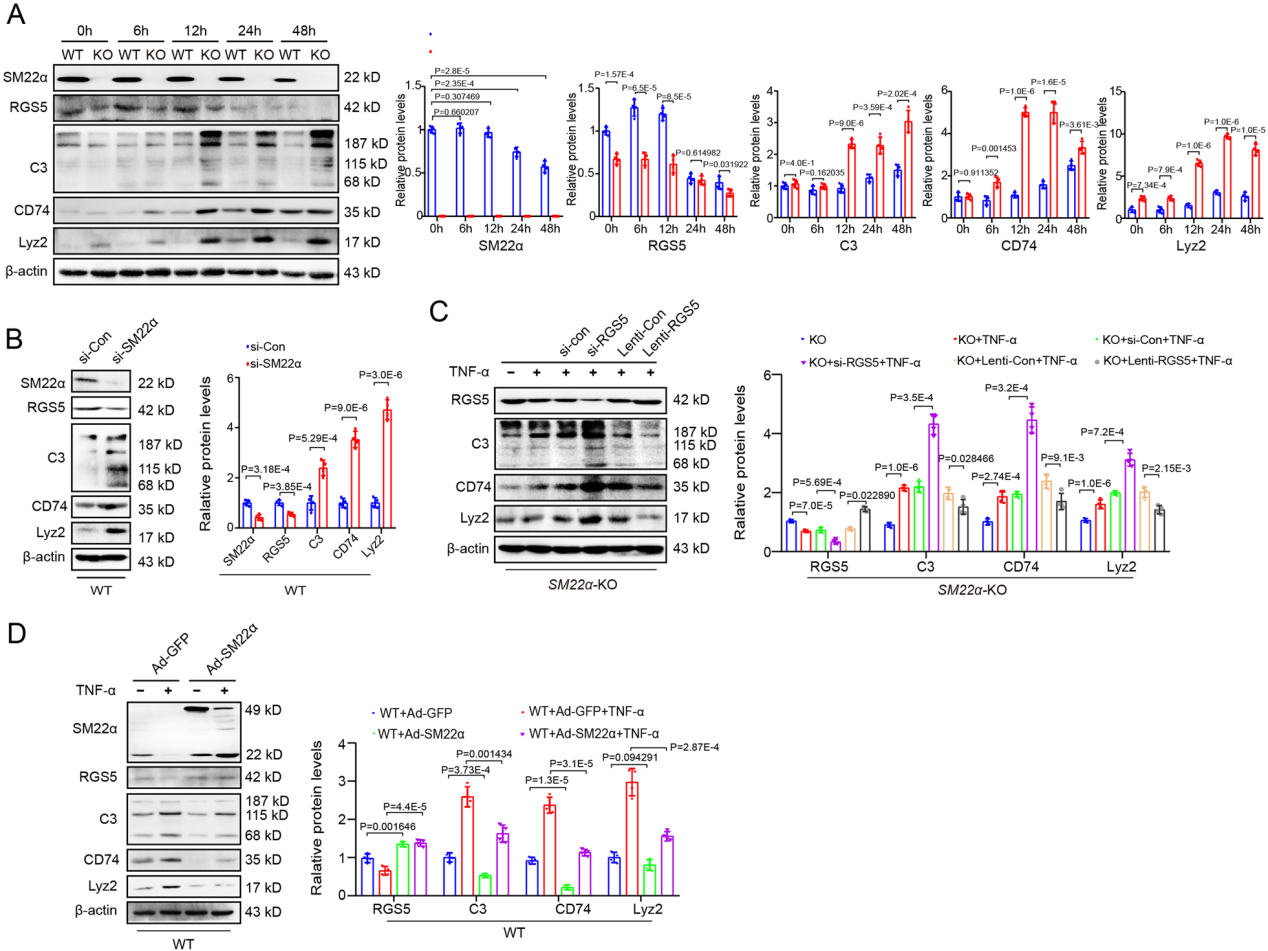
SM22α acts additively and synergistically with RGS5 to suppress VSMC inflammation. (**A**) Western blot analysis and quantification of SM22α, RGS5 and macrophage-like markers (C3, CD74 and Lyz2) in WT and *SM22α*-KO VSMCs stimulated with TNFα for 0, 6, 12, 24 and 48 h, respectively. (**B**) Protein levels of SM22α, RGS5 and macrophage-like markers (C3, CD74 and Lyz2) in WT VSMCs transfected with si-Con (50 nM) or si-SM22α (50 nM). (**C**) Western blot analysis and quantification of RGS5 and macrophage-like markers (C3, CD74 and Lyz2) in *SM22α*-KO VSMCs transfected with RGS5-siRNA (si-Con or si-RGS5) or RGS5-lentivirus (Lenti-RGS5 or vector control) followed by TNF-α treatment. (**D**) Western blot analysis and quantification of SM22α, RGS5 and macrophage-like markers (C3, CD74 and Lyz2) in WT VSMCs transfected with Ad-GFP and Ad-SM22α with or without TNF-α stimulation. The data were presented as the mean ± SD

SM22α loss is a prominent marker of phenotypic switching of VSMCs [19, 20]. *SM22α*-KO mice display a higher risk of atherosclerosis [21, 22]. We showed that *SM22α*-KO VSMCs had a lower level of RGS5 protein and higher macrophage-like markers upon TNF-α treatment compared to WT cells (Fig. 4A). Furthermore, although knockdown of SM22α, as like RGS5 silencing, could induce the inflammatory response of VSMCs, a much stronger inductive effect was observed when both were applied (Fig. 4B, C). RGS5 overexpression attenuated the inflammation of *SM22α*-KO VSMCs to some extent (Fig. 4C). Similarly, overexpression of SM22α eliminated the down-regulation of RGS5 expression induced by TNF-α treatment, therefore attenuating the level of VSMC inflammatory markers in WT VSMCs (Fig. 4D), providing further support for SM22α additively and synergistically with RGS5 to suppress VSMC pro-inflammatory phenotypic switching.

### High RGS5 expression is associated with increased tumor vasculature in the patients with breast cancer

**Fig. 5:**
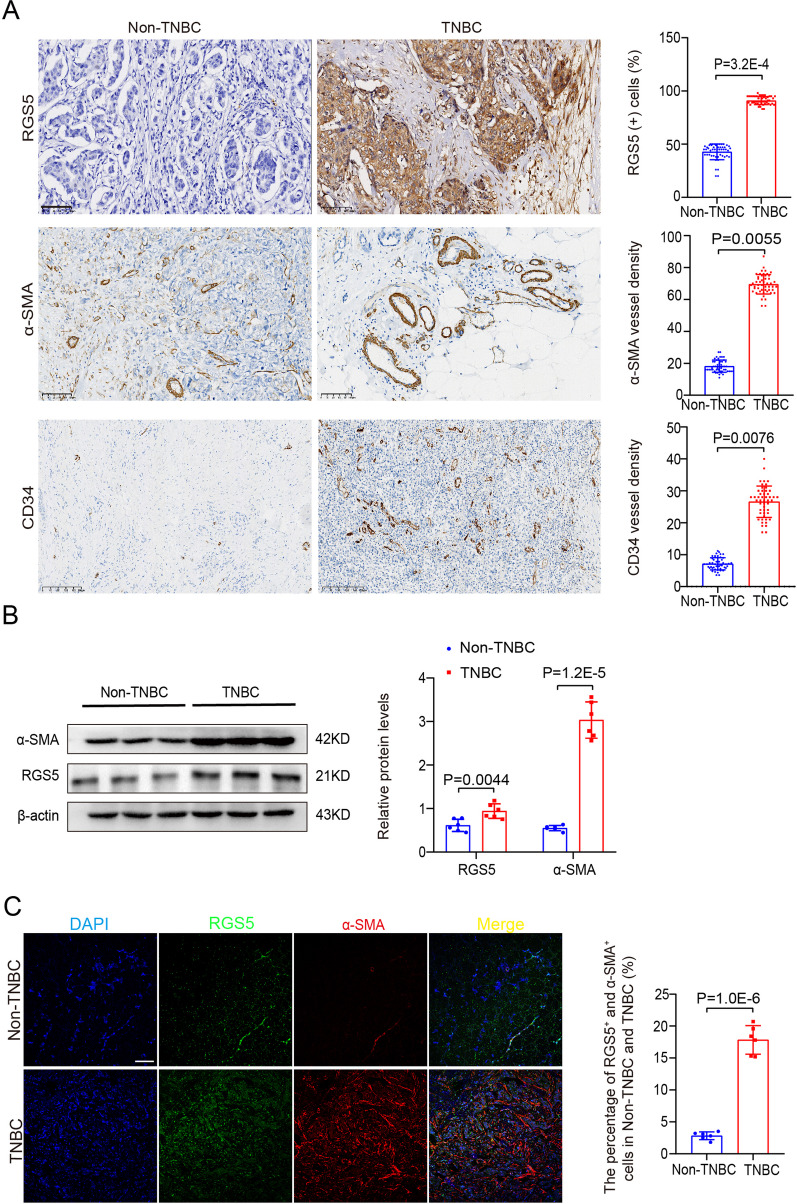
High RGS5 expression is associated with increased tumor vasculature in the patients with breast cancer. (**A**) Representative immunohistochemical staining for RGS5, α-SMA and CD34 expression in TNBC and adjacent normal tissues. Scale bar = 100 μm. Right: Quantification of the immunohistochemistry (n = 50). (**B**) Western blot and quantification of α-SMA and RGS5 in TNBC tissues. (**C**) Representative immunofluorescence images of RGS5 and α-SMA in TNBC tissues. Scale bar = 100 μm. The data were presented as the mean ± SD

Remodeling of tumor blood vessels supports their survival and expansion and provides accessibility to the vasculature and a route of transport for metastasizing tumor cells. Angiogenesis is also an essential step in the early steps of hematogenous metastasis formation by enabling tumor cells to connect to the preexistent vasculature. Furthermore, cellular and/or molecular changes to pre-existing vessels may represent subtle pre-metastatic alterations to the vasculature. To determine the relationship between RGS5 expression and remodeling of the vasculature in the tumors, we examined the sections of human breast cancer tissues by immunohistochemical staining. We showed that higher RGS5 expression and blood vessel network co-existed in the triple-negative breast cancers (TNBCs) that constituted the most frequent type of invasive breast cancer. The number of arteriole (α-SMA^+^) and capillaries (CD34^+^) in TNBCs was more than that of non-TNBCs (Fig. 5A). Increased expression of RGS5 and α-SMA in TNBCs were further validated by Western blot (Fig. 5B). Immunofluorescence staining also showed that RGS5 was mainly enriched in the wall of and around the arterioles in TNBC tissues (Fig. 5C), suggesting that RGS5 expression in VSMCs increased in the context of the tumor microenvironment, which may be involved in remodeling of the vasculature in tumor.

### Tumor microenvironment modulates the pro-inflammatory phenotypic switching of VSMCs by tumor-derived RGS5

**Fig. 6:**
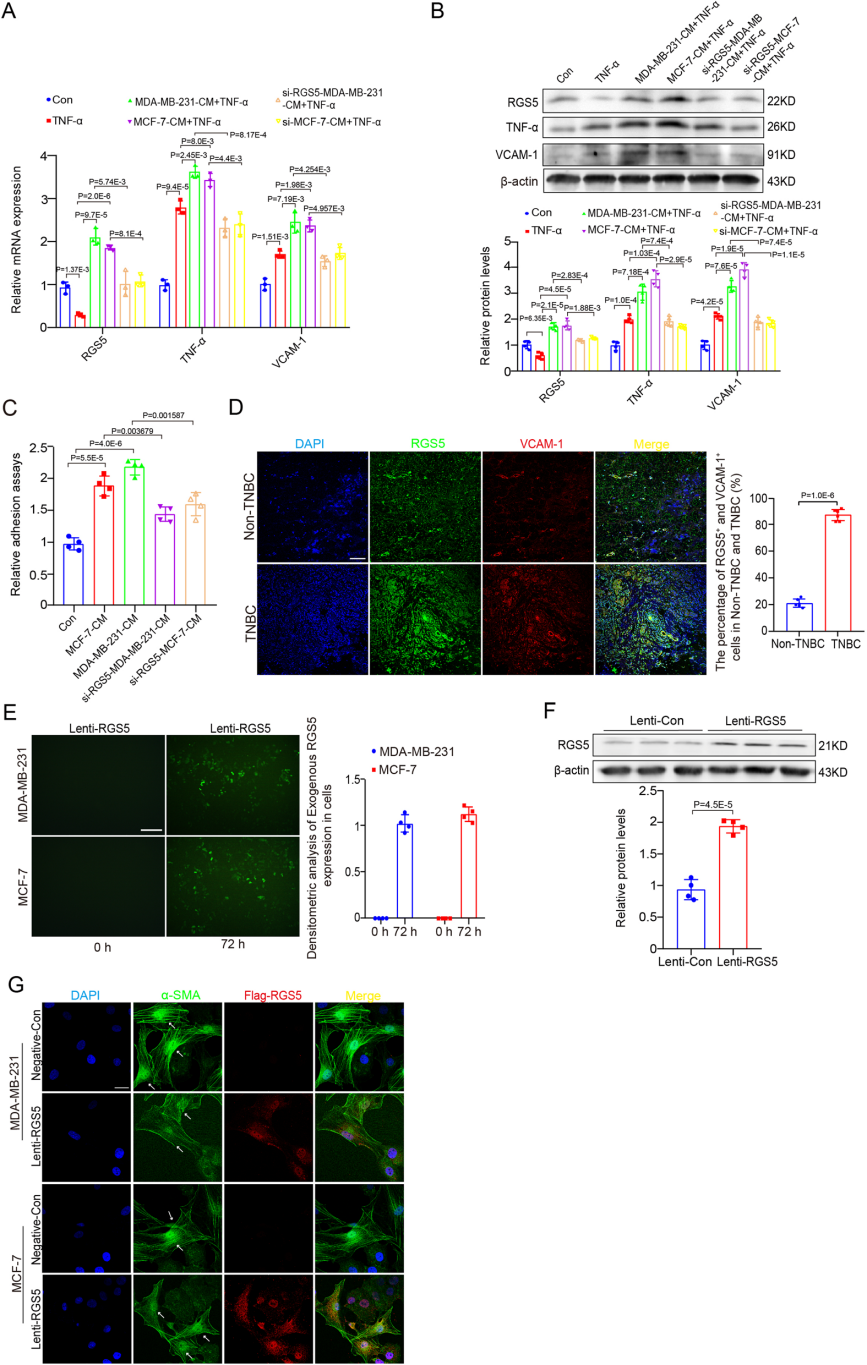
Tumor microenvironment modulates the pro-inflammatory phenotypic switching of VSMCs by tumor-derived RGS5. (**A, B**) qRT-PCR and Western Blot analysis the expression of RGS5, TNF-α and VCAM-1 in BC-CM-pre-treated, untreated VSMCs (Con) or RGS5 knockdown BC-CM-pre-treated VSMCs for 24 h followed 24 h of TNF-α stimulation. (**C**) The fluorescent intensity quantification of BC-CM-pre-treated, untreated VSMCs (Con) or RGS5 knockdown BC-CM-pre-treated VSMCs for 24 h followed calcein-AM-labeled RAW264.7 cell adhesion for 30 min. (**D**) Representative immunofluorescence images of RGS5 with the pro-inflammatory markers VCAM-1 in TNBCs and Non-TNBC. Scale bar = 100 μm. (**E**) Lenti-RGS5 was infected into the breast cancer cells. Scale bar = 100 μm. (**F**) Western blot analysis and quantification of RGS5 expression in the tumor cells after Lenti-RGS5 infection. (**G**) Representative immunofluorescence images of VSMCs co-incubation with the tumor cells infected with Lenti-RGS5 or not. VSMCs are marked with white arrows. Scale bar = 25 μm. The data were presented as the mean ± SD

VSMC phenotypic switching plays a key role in the formation of arteriole, which improves tissue blood supply. To determine the effect of the tumor microenvironment on VSMC phenotype, VSMCs were treated with the breast cancer cells MDA-MB-231 and MCF-7-conditioned media (BC-CM). qRT-PCR and Western blot showed that BC-CM induced the expression of RGS5, TNF-α and VCAM-1 at mRNA and protein levels in VSMCs (Fig. 6A, B), representing a pro-inflammatory phenotype, different from TNF-α treatment alone, which reduced RGS5 expression in VSMCs with pro-inflammatory phenotype. Notably, the BC-CM-treated VSMCs also exhibited enhanced adhesion activity to macrophages compared with the control (Fig. 6C). In contrast, VSMCs were treated with BC-CM from RGS5 knockdown breast cancer cells reversed the upregulation of TNF-α, VCAM-1 and macrophages adhesion (Fig. 6A-C). These findings suggest that BC-CM confers VSMCs to exhibit a pro-inflammatory adhesion phenotype that is prone to tumor invasion. Moreover, the effect of RGS5 on pro-inflammatory phenotype was supported by enhanced co-location of RGS5 with the pro-inflammatory markers VCAM-1 in TNBCs (Fig. 6D). To further validate whether the tumor-derived RGS5 switches its function from inhibiting inflammation to promoting inflammation, Lenti-RGS5 was infected into and expressed in the breast cancer cells (Fig. 6E, F). And then, the Lenti-RGS5-infected cancer cells were co-cultured with VSMCs. Immunofluorescence staining showed that Flag-RGS5 derived from Lenti-RGS5-infected cancer cells was detected in α-SMA^+^ VSMCs after co-incubation for 48 h (Fig. 6G), indicating that the tumor-derived RGS5 was transferred into VSMCs. Collectively, these data suggest that tumor microenvironment induces the pro-inflammatory phenotypic switching of VSMCs by tumor-derived RGS5.

## Discussion and conclusion

Blood vessels supply every part of a living organism with oxygen and nutrients, for which reason the establishment of a mature organized vascular network is fundamental for tissue homeostasis. However, tumor vasculature, the blood vessel network supplying a growing tumor with nutrients such as oxygen or glucose, is in many respects different from the hierarchically organized arterio-venous blood vessel network in normal tissues. The tumor has to switch to an angiogenic phenotype and to induce the development of new blood vessels. This process is regulated by a variety of pro- and antiangiogenic factors. In this study, we identified that RGS5 protected against inflammation and pyroptosis of VSMCs and reduced infiltration of inflammatory immune cells, thereby inhibiting vascular remodeling and normalizing vascular repair. However, in the context of the tumor microenvironment, RGS5 shifted its function from inhibiting inflammation to inducing the pro-inflammatory phenotypic switching of VSMCs, which enhanced the ability to adhesion with the tumor cells and favors tumor metastasis.

The blood vessels supplying tumors are strikingly heterogeneous and differ from their normal counterparts with respect to organization, structure, and function. Unlike normal vessels, the tumor vasculature is structurally and functionally abnormal, with defects in the endothelium, pericyte coverage, and basement membrane [23]. Tumor-induced angiogenesis has been well studied [24]. However, the coverage of VSMCs and pericytes is necessary for the formation of the blood vessel network that carries a blood flow [25]. Genomic alterations shape cell phenotypes and the structure of tumor ecosystems. The recent study revealed distinct combinations of cell phenotypes and cell–cell interactions were associated with genomic subtypes of breast cancer [26]. Furthermore, VSMCs have identified as a cell phenotype in breast tumors and were associated with favorable prognosis [26], suggesting that VSMCs are involved in formation of both the composition and architecture of breast tumor ecosystems. However, the feature of VSMC phenotypes in the tumor tissues is poorly defined.

Vascularization is required for tumor growth and metastasis, and constitutes a key step in the control of cancer progression [27]. The previous study indicated that overexpression of RGS5 promoted tumor metastasis by inducing epithelial-mesenchymal transition [28]. In RGS5-transfected VSMCs and fibroblasts, RGS5 could attenuate the signaling triggered by proteins mediating vascular functions such as angiotensin II (AngII), endothelin-1 (ET-1), and sphingosine-1-phosphate (S1P) [13, 29], suggesting a possible involvement of RGS5 in blood vessel maturation and homeostasis. Furthermore, RGS5 acts as a novel marker of cancer vasculature and the expression of RGS5 is elicited and sustained by a milieu of factors typical of the proangiogenic tumor environment [30]. In this study, we provided evidence that intrinsic RGS5 expression defended against chronic sustained inflammation and deleterious remodeling of the artery via modulating local immune homeostasis. RGS5 overexpression in VSMCs showed hampered inflammatory progression and reduced pyroptosis in vitro and in vivo, thereby resolving inflammation and retarding the vascular remodeling. Given previous finding that SM22α inhibited NF-κB activation and vascular inflammation [31, 32], we validated that RGS5 acted additively and synergistically with SM22α to suppress VSMC inflammation. The findings of loss-of-function and gain-of-function provide explicit evidence that RGS5, as a regulator of VSMC phenotypic switching, is necessary and sufficient for maintaining VSMC homeostasis and repair process in the setting of vascular injury. Intriguingly, RGS5 expression was increased in TNBCs tissues and in the tumor blood vessels, accompanied with an extensive vascular network. VSMCs treated with BC-CM displayed enhanced pro-inflammatory phenotype and higher adherent with macrophages. Notably, the pro-inflammatory VSMCs triggered by BC-CM highly expressed RGS5, different from the above mentioned the pro-inflammatory phenotype that had a reduced RGS5. Importantly, tumor-derived RGS5 could be transferred into VSMCs, which may explain increased RGS5 in pro-inflammatory VSMCs triggered by BC-CM. Taken together, these findings suggest that tumor microenvironment shifts the function of RGS5 from anti-inflammation to pro-inflammation and induces the pro-inflammatory phenotype of VSMCs that is favorable for tumor metastasis. The tumor-derived RGS5 may be responsible for the aberrant VSMC pro-inflammatory phenotype.

### Supplementary Information

**Additional file 1:** Table.

**Additional file 2:** Supplementary figures.

## Data Availability

The authors declare that all supporting data and methods are available within the article and its online supplementary files.
