# Supplementary material for: RGS5 maintaining vascular homeostasis is altered by the tumor microenvironment
Source: Biol Direct. 2023 Nov 20;18:78. doi: 10.1186/s13062-023-00437-y (PMC10662775; doi:10.1186/s13062-023-00437-y)
Supplement: Supplementary file 1 — Additional file 1: Table. [file 13062_2023_437_MOESM1_ESM.docx]

**Table S1.** List of specific primer sequences used for qRT-PCR.

| **Species** | **Gene** | **Forward primer** | **Reverse primer** |
| --- | --- | --- | --- |
| Mouse | C3 | TCGGCATAGAGAAGAGGCAAGAGG | TTGTTGAAGGCAGCATAGGCAGAG |
| Mouse | CD74 | AGATGCGGATGGCTACTCC | TCATGTTGCCGTACTTGGTAAC |
| Mouse | LYZ2 | GAGCTGTGAATGCCTGTGGGATC | GACAGTGTGCTCGCCATGCC |
| Mouse | RGS5 | ACAAACTTCTCCAGAGCAACTACGG | CACAGGCAACCCAGAACTCAAGG |
| Mouse | VCAM-1 | AGTTGGGGATTCGGTTGTTCT | CCCCTCATTCCTTACCACCC |
| Mouse | TNF-α | CTTCCAGAACTCCAGGCGGT | ACTTGGTGGTTTGCTACGACG |
| Mouse | β-actin | CCACCATGTACCCAGGCATT | AGGGTGTAAAACGCAGCTCA |
