# Supplementary material for: RGS5 maintaining vascular homeostasis is altered by the tumor microenvironment
Source: Biol Direct. 2023 Nov 20;18:78. doi: 10.1186/s13062-023-00437-y (PMC10662775; doi:10.1186/s13062-023-00437-y)
Supplement: Supplementary file 2 — Additional file 2: Supplementary figures. [file 13062_2023_437_MOESM2_ESM.docx]

**
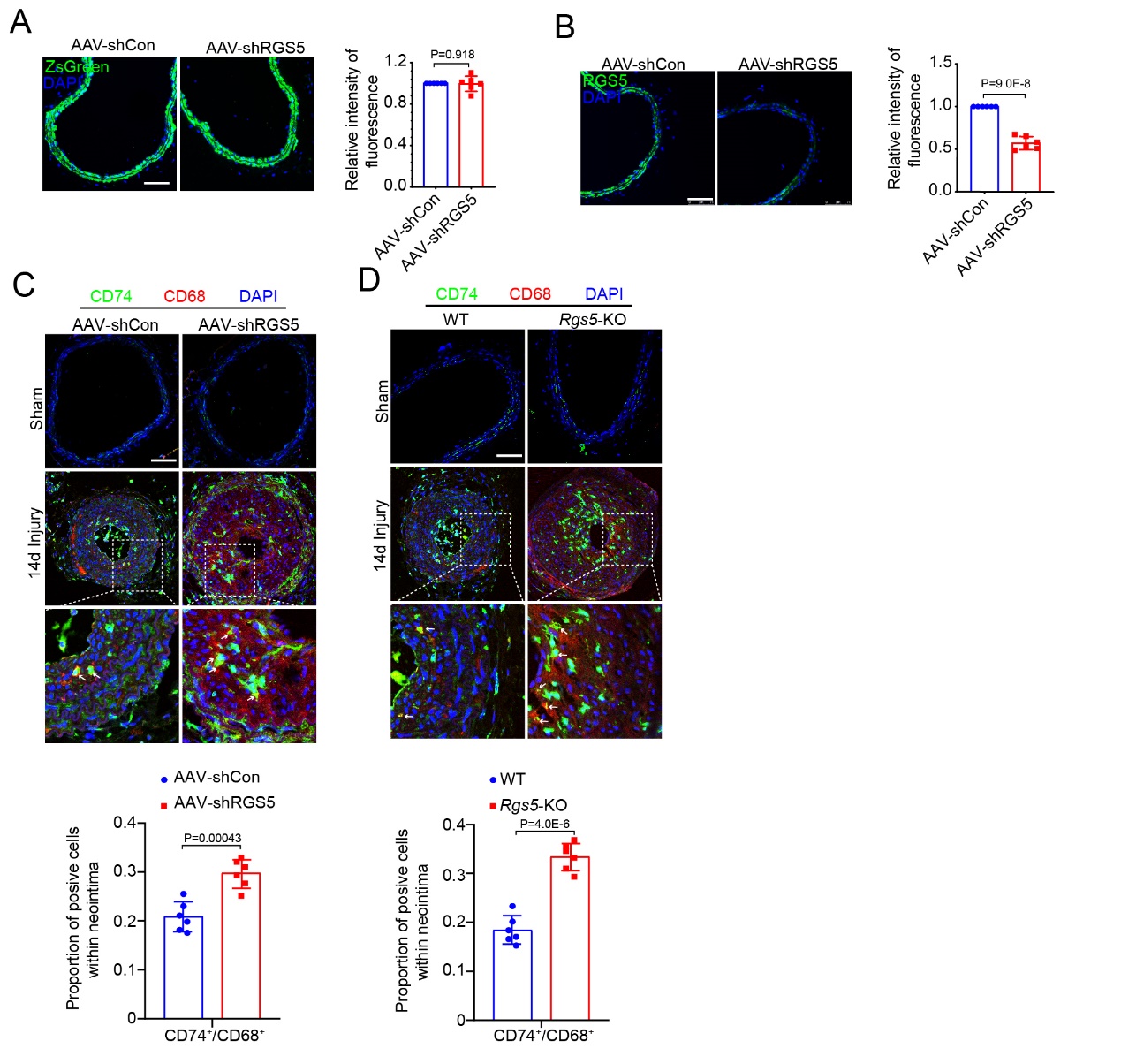
**

**Fig.S1 RGS5 knockdown in vivo. (A, B)** Left: Representative immunofluorescence images of ZsGreen (A) or RGS5 (B) stained in mouse carotid artery transfected with AAV-shRGS5 and AAV-shCon for 4 weeks. Nucleuses stained by DAPI were indicated in blue. Scale bar=75 μm. Right: The [relative fluorescence intensity](javascript:;) in each group. **(C, D)** Representative immunofluorescence images of CD74 and CD68 in sham-operated and ligated carotid arteries at day 14 post surgery of 10-week-old male WT mice intravenous infected with AAV-shRGS5 or 10-week-old male *Rgs5*-KO mice and WT littermates. Scale bar=75 µm. Lower: Quantitative analysis of the proportion of CD74^+^/CD68^+^ cells. The data were presented as the mean±SD.

**
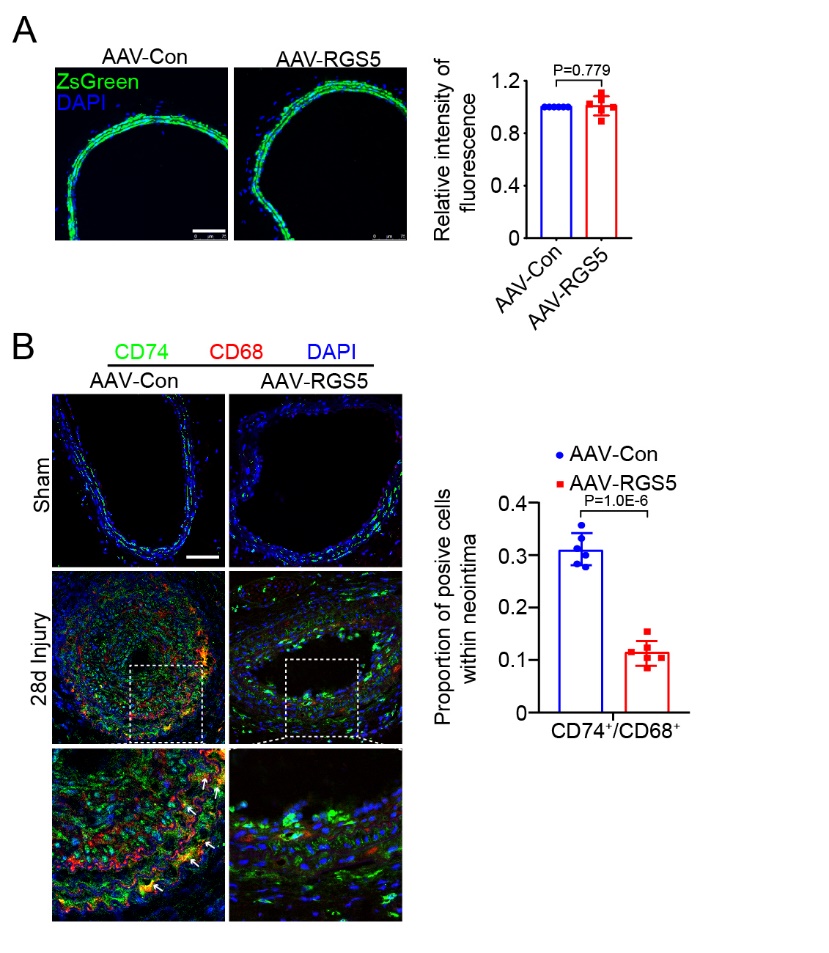
**

**Fig.S2 RGS5 overexpression in vivo. (A)** Left: Representative immunofluorescence images of ZsGreen (green) stained in mouse carotid artery transfected with AAV-Con and AAV-RGS5 for 4 weeks. Nucleuses stained by DAPI were indicated in blue. Scale bar=75 μm. Right: The relative fluorescence intensity in each group. **(B)** Representative cross sections of immunofluorescence images of CD74 and CD68 in sham-operated and ligated carotid arteries at day 28 post surgery of 10-week-old male WT mice intravenous infected with AAV-Con and AAV-RGS5. Scale bar=75 µm. Right: Quantitative analysis of the proportion of CD74^+^/CD68^+^ cells in each group. The data were presented as the mean±SD.
